# An analytic hierarchy process analysis for reinforcing doctor–patient communication

**DOI:** 10.1186/s12875-023-01972-3

**Published:** 2023-01-21

**Authors:** Sewon Park, Han-Kyoul Kim, Munjae Lee

**Affiliations:** 1 Department of Medical Humanities and Social Medicine, Ajou University School of Medicine, Suwon, South Korea; 2 Department of Rehabilitation Medicine, Seoul National University Hospital, Seoul, South Korea; 3 National Traffic Injury Rehabilitation Research Institute, National Traffic Injury Rehabilitation Hospital, Yang- Pyeong, South Korea; 4 Medical Research Collaborating Center, Ajou Research Institute for Innovative Medicine, Ajou University Medical Center, Suwon, South Korea

**Keywords:** Health communication, Patient-doctor communication, Doctor-Patient relationship, Quality of medical service, AHP analysis

## Abstract

**Background:**

As the health paradigm shifts toward patient-centeredness, patients can actively participate in their own treatment. However, there is still a unilateral aspect of doctor-patient communication, so it is necessary to specify obstacles between doctors and patients. Therefore, this study attempted to extract obstacles that block doctor-patient communication and to analyze differences in perception of doctor-patient communication.

**Methods:**

A total of 35 questionnaires composed of brainstorming for the study were distributed, and a total of 21 questionnaires were used for analysis. The collected data was analyzed by AHP using dress ver 17.0.

**Results:**

As a result of the study, doctors ranked the priority of health communication in the order of professionalism, reliability, fairness, communication, and psychologically. On the other hand, for patients, the priority factors of health communication were communication, fairness, professionalism, reliability, and psychologically.

**Conclusion:**

In order to improve the quality of health communication between doctors and patients, doctors will be able to communicate from the patient’s point of view and strengthen communication with patients by providing consistent medical services and patients need to trust the doctor and patients need to trust their doctors and participate in the medical process faithfully.

**Supplementary Information:**

The online version contains supplementary material available at 10.1186/s12875-023-01972-3.

## Background

In a medical institution, the treatment of diseases starts with communication between doctors and patients; interactions between doctors and patients take place through communication. A doctor comes to infer a patient’s disease status through the patient’s explanations. The patient may better understand his or her own condition through the doctor’s explanation and learn about the treatment method and other key information. Indeed, doctors set the directions in medical judgments and treatments, based on information obtained via conversations with their patients. Patients are also more likely to follow their doctor’s diagnosis and a prescription if the doctor positively explains the treatment process. Accordingly, effective communication between doctors and patients can be seen as important to enable accurate diagnoses and treatments [1, 2]. Doctors may establish treatment plans through communication with patients, and patients come to receive medical services through interactions in the treatment process. However, if communication between doctors and patients is not smooth, a patient may distrust the doctor’s medical service, and adherence may decrease. In other words, if communication between doctors and patients is not satisfactory, the quality of medical services can deteriorate, and the economic costs, including medical expenses and the treatment period, that the patient has to bear would increase [2, 3].

As the medical paradigm shifts to patient-centered medicine, patients have been made to make decisions on their treatment to actively participate in the treatment [4]. If communication between doctors and patients increases due to the provision of patient-centered medical services, this may not only relieve a patient’s symptoms but also reduce the uncertainties, patient’s stress and depression, medical accident risks, etc. that come with treatment, all of which may be caused by a lack of communication. In addition, unnecessary examinations and treatments would be reduced, positively affecting the medical process, such as through reduced medical costs and so forth, and treatment results [5–7]. In particular, as aging is progressing globally and the number of chronically ill is increasing around the world, not only have medical expenses increased but also the personal burden on health care. Chronic diseases are a major cause of death and require continuous treatment and management. As doctor–patient communication is increasingly reinforced, patients will be able to understand the treatment process and alleviate their diseases through effective health management. That is, if medical information is exchanged by improving the communication between doctors and patients, doctors would be able to provide treatment methods suitable for each patient. Thus, we expect that the adherence of patients with the diagnoses of doctors will be enhanced, thereby resulting in improved treatment results [8–10].

As such, the importance of doctor–patient communication is being emphasized, but there is an aspect that doctor–patient communication is still unilaterally performed. Research on health communication has already been conducted focusing on the measurement of health communication, including measures for doctor–patient communication and doctor–patient mutual understanding, among others; however, there is currently insufficient research that materializes the obstacles to doctor–patient communication and that analyzes measures to reinforce their mutual communication [11–13]. Accordingly, in this study, we intend to derive the priorities for smooth communication between doctors and patients through an AHP (Analytic Hierarchy Process) analysis. This study aims to extract obstacles that obstruct doctor–patient communication and then analyze differences in perception regarding doctor–patient communication. We intend to synthetically derive the doctor–patient’s viewpoint of health communication and present a strategy to improve the doctor–patient relationship.

## Methods

### Research process

**Fig. 1:**
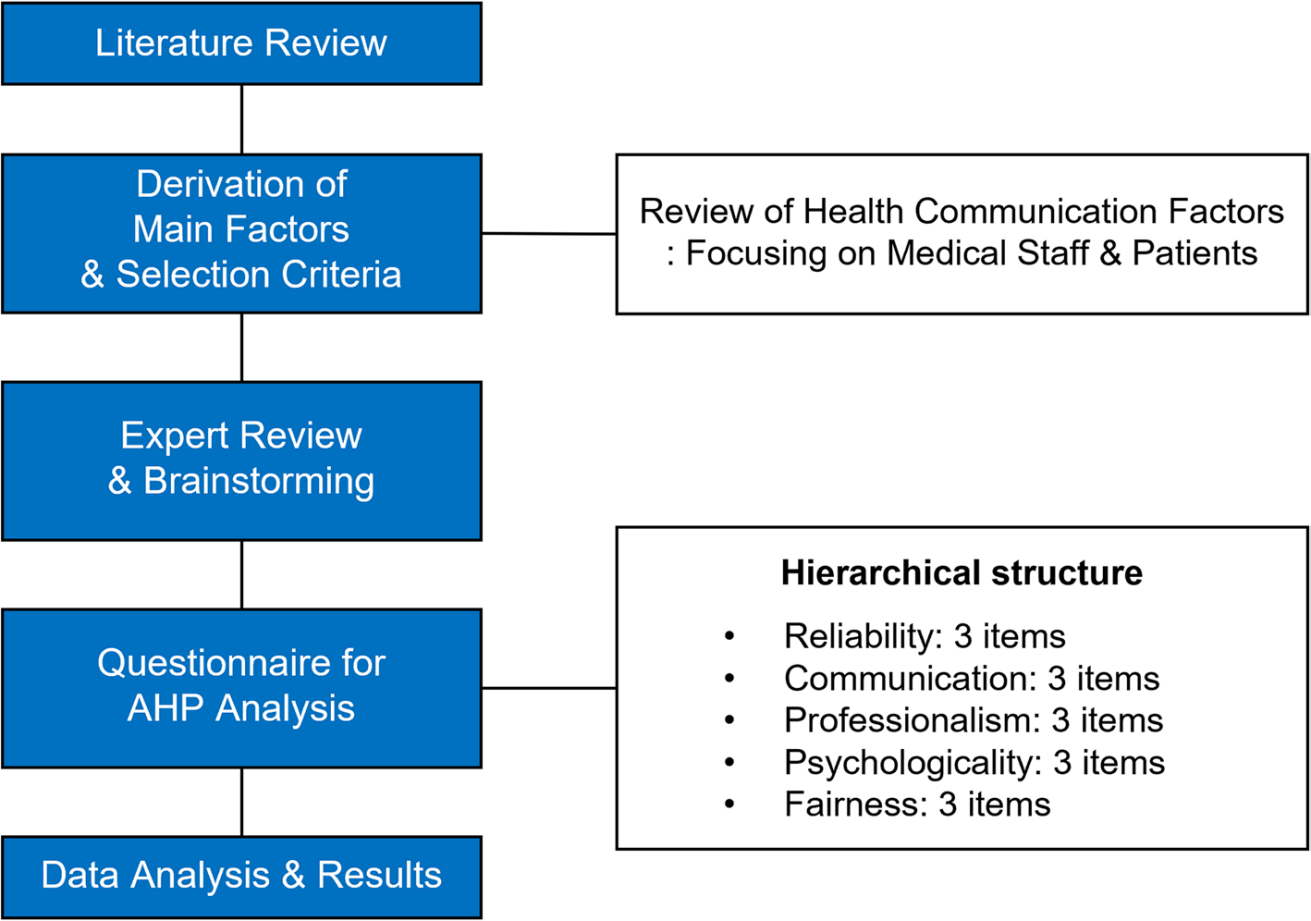
An overview of the methodology

In this study, AHP analysis was conducted to identify the factors affecting the reinforcement of doctor-patient communication. The detailed methodology is shown in Fig. 1. First, major factors were selected from previous studies that had analyzed mutual communication, general communication, and other kinds of communication. between doctors and patients, and then each factor was defined based on these previous studies. Furthermore, medical staff, including doctors and nurses, and six patients were selected to investigate whether the extracted factors may affect the improvement of doctor–patient communication. Lastly, a questionnaire was prepared by reviewing previous studies and brainstorming each factor extracted mainly from experts. Based on this, factors affecting doctor-patient communication were defined as reliability, communication, professionalism, psychologically, and fairness. The relative weight of the indicator was evaluated using AHP.

### Research framework and variables

The Physician–Patient Relationship Scale proposed by Park Jin-young (2014) was utilized as the main factor for doctor–patient communication [14]. The Physician–Patient Relationship Scale represents the perception difference and mutual understanding between doctors and patients. It had been created to set the direction to improve the relationship between doctors and patients. The doctor–patient relationship comprises nine elements: reliability, fidelity, satisfiability, interchangeability, social contribution, mutual intelligibility, professionalism, fairness, and rapport. Among these, reliability, mutual intelligibility, and fairness, derived through a confirmatory factor analysis, were utilized. In addition, the service quality SERVQUAL measurement model proposed by Parasuraman et al. (1988) was used [15]. The major dimensions of SERVQUAL’s service quality include reliability, responsiveness, guaranteedness, empathy, and tangibility. Among the 10 component factors of the measurement model, reliability, capability, and understandability were utilized. Since ability has the same meaning as professionalism, these were unified into professionalism. Understandability refers to an individual’s consideration to understand a customer’s needs and acts to stabilize the customer’s psychological state; the name of this factor was changed to psychologically. Accordingly, reliability, mutual intelligibility, professionalism, psychologically, and fairness were derived as the main factors for the AHP analysis to reinforce the doctor–patient communication. The definitions for each factor are provided in Table 1.

**Table 1:** Definition of main factors

Factor	Definition
Reliability	Doctors’ and patients’ confidence or trust in each other amid treatment
Communication	Doctors and patients exchange opinions freely
Professionalism	Reliability of doctors’ and patients’ abilities in the treatment process
Psychologically	Consideration and tight-knitedness between doctors and patients
Fairness	Consistency in medical care services

In previous studies on the quality of medical services, reliability meant that the provided medical services were reliable and also referred to the ability to reliably deliver these services to patients. It exhibits the state of convincing each other, as physicians provide reliable medical services for patients and the patients faithfully accept medical services provided by physicians. Buyukozkan et al. [16] evaluated the quality of medical services through an AHP analysis of the elements of SERVQUAL to determine the priorities in providing medical services. As a result, empathy and reliability were found to be important in evaluating the quality of medical services. Reliability was measured by the accuracy and consistency of the given information and the protection of patient information, among others. By using this, the sub-items of reliability were composed of the conformity of the doctor’s words and actions, the provision of accurate information appropriate to the patient’s needs and level, and the patient’s sincere participation in treatment.

Mutual intelligibility refers to the state that a physician and a patient exchange opinions with each other and reflect these in their actions. It represents a communication quality beyond the two-way communication between doctors and patients, including both verbal and non-verbal communication. For the sub-items to measure mutual-intelligibility, the Doctor’s and Patient’s Intent Assessment Tool developed by Makoul et al. (2007) was utilized [17]. The measurement items for this assessment tool included, among others, “He or she paid attention to me,” “He or she made sure whether I understood everything,” and “He or she encouraged me to ask questions.” Through this tool, the sub-items of mutual intelligibility were composed of “The doctor listens to the patient’s opinion,” “The doctor discusses the treatment process with the patient,” and “The doctor checks whether the patient has perceived the explained content.”

In existing health communication studies, professionalism appears to be a factor that has the greatest influence on the quality of doctor–patient relationships. Professionalism is defined as an objective indicator of whether the physician has the ability to provide accurate and effective treatment for the patient. Furthermore, as interest in health has recently increased and information and communication technology has developed, it is changing into a structure in which patients can easily receive medical information as well. Therefore, at times, the patient’s understanding of the treatment process is also included in professionalism. By utilizing the professionalism evaluation items used by Marley et al. (2004) to analyze the quality of medical services, the sub-items of professionalism were composed of “the accuracy of the doctor’s diagnosis and prescription,” “the doctor has the medical knowledge at a professional level and the latest information,” and “the patient’s high awareness of the treatment process” [18].

Psychologically acts to reduce patient anxiety and concerns by creating a sense of psychological trust between doctors and patients and to increase patient satisfaction with doctors’ treatment. In addition, doctors can provide smooth medical services by creating a comfortable atmosphere to increase patients’ understanding of the treatment process. As the sub-item to measure psychologically, the research findings of Jagosh et al. (2011), who studied the importance of listening between doctors and patients and their various functions, were utilized. Jagosh et al. (2011) stated that doctors’ listening may reinforce the relationship between doctors and patients to greatly contribute to the treatment effect [19]. In particular, they asserted that the non-verbal elements of a doctor’s communication further facilitates communication between the doctor and the patient and that positive listening by the doctor can stabilize the patient’s psychological state and thereby make the patient actively communicate his own situation to the doctor. Using these research results, the sub-items of psychologically consisted of “the doctor creates a comfortable atmosphere to listen to my story,” “I trust and rely on the doctor,” and “the kindness of the doctor.”

**Fig. 2:**
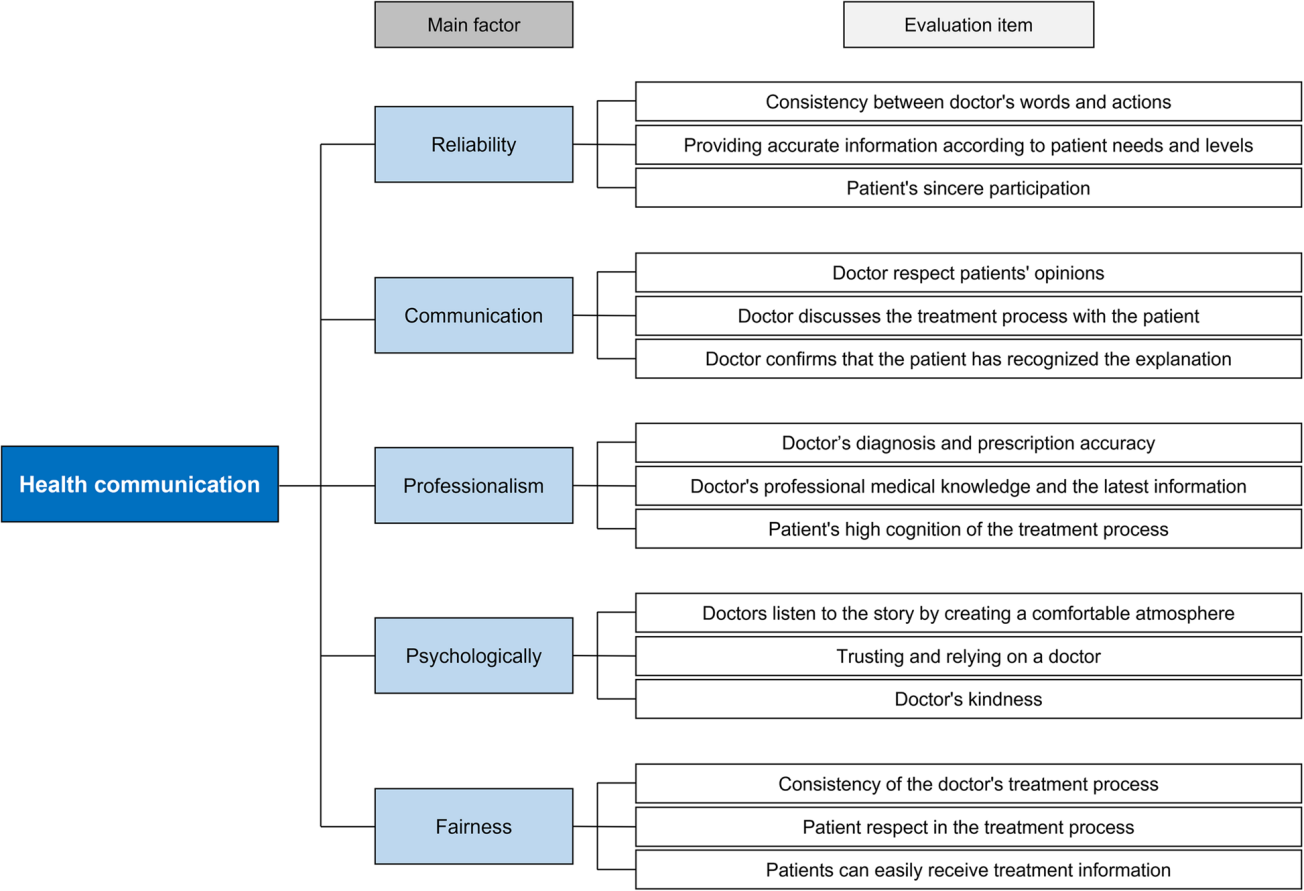
Research framework

Fairness means that physicians treat all patients fairly, which is also included under the concept of medical ethics contained in the Hippocratic Oath. Liang et al. [4, 20] used a model developed to measure the perception of doctor–patient relationships and patient satisfaction. They stated that the closer the relationship between doctors and patients, the higher the patient satisfaction and the higher the patient’s awareness of service fairness becomes as well. In addition, they classified fairness into distributed fairness (the fairness of the cost paid by the patient and of medical services), procedural fairness (when the staff provides the medical service requested by the patient in a reasonable manner), interaction fairness (the attitude respecting the patient), and information fairness (easily provided treatment information). Using this, the sub-items of fairness were composed of “the consistency of the doctor’s treatment process,” “the attitude respecting the patient when performing treatment,” and “patients can easily receive treatment information at the hospital.” (See Table 2; Fig. 2).

**Table 2:** Health communication evaluation item

Main factor	Evaluation item	Related References
Reliability	Consistency between doctor’s words and actions	[16, 21]
	Providing accurate information according to patient needs and levels	
	Patient’s sincere participation	
Communication	Doctor respect patients’ opinions	[17, 22]
	Doctor discusses the treatment process with the patient	
	Doctor confirms that the patient has recognized the explanation	
Professionalism	Doctor’s diagnosis and prescription accuracy	[18, 23]
	Doctor’s professional medical knowledge and the latest information	
	Patient’s high cognition of the treatment process	
Psychologically	Doctors listen to the story by creating a comfortable atmosphere	[19, 24, 25]
	Trusting and relying on a doctor	
	Doctor’s kindness	
Fairness	Consistency of the doctor’s treatment process	[4, 20]
	Patient respect in the treatment process	
	Patients can easily receive treatment information	

### Analysis method

AHP was developed by Saaty in the 1980s and is used to determine the priority and importance of elements constituting the decision-making hierarchy. It is based on MCDM (Multi-Criteria Decision Making) and determines the importance of decision-makers judgments based on weights derived from pairwise comparisons between elements. In AHP, the pairwise comparison is performed by performing a comparative evaluation based on the weight of each element in the lower tier compared to other lower elements based on the element in the upper tier [26, 27]. Through this hierarchical analysis, it is possible to grasp the relative importance of elements in the immediate lower tier from the perspective of one upper tier. To more specifically analyze the importance of the five upper classes classified in this study, 15 lower classes were constructed to explain the upper classes, and priorities were derived through pairwise comparison. In other words, health communication was constituted as the highest layer, and reliability, communication, professionalism, psychologically, and fairness were set as the then the upper tier, and a hierarchical structure was established by subdividing evaluation criteria that are representative of health communication in each lower layer.

AHP is flexible in handling complex decision problems, and it can overcome the limitations of quantitative analysis in that it prioritizes problems of different importance according to subjective preferences, such as doctor-patient communication, by using expert knowledge. AHP analysis basically goes through the following steps [28].

- Step 1: Establish a final goal and list related elements to build a hierarchy based on interrelated criteria.
- Step 2: Pairwise comparison is performed for each layer to compare the weights of each element. Using a score from 1 to 9, which is the basic scale of AHP, each pair is compared according to the expert’s judgment and the importance is judged (See Table 3).

**Table 3:** Scale for Pairwise Comparison

Number value	Importance exposition
1	Equal importance
3	Moderately more importance
5	Strongly importance
7	Very Strongly more importance
9	Extremely more importance
2,4,6,8	Intermediate values between adjacent judgments

- Step 3: Calculate the pairwise comparison value answered by the expert group and calculate the relative weight for each element. In pairwise comparison, when evaluation factor $i$ is compared with other factor $j$, the importance can be seen as ${a}_{ij}$. The pairwise comparison matrix is composed of $An\times n$, and the range of ${\omega }_{i}$ ranges from 1 to 9, indicating the relative importance of evaluation factors. The specific expression for the pairwise comparison matrix is as follows.

$$A=\left[a_{ij}\right]=\begin{array}{cccc}a_{11}&a_{12}&\dots&a_{1n}\\a_{21}&a_{22}&\dots&a_{2n}\\\vdots &\vdots&\ddots&\vdots\\a_{n1}&a_{n2}&\dots&a_{nn}\end{array}=\left\{\begin{array}{cccc}\frac{\omega_1}{\omega_1}&\frac{\omega_1}{\omega_2}&\cdots&\frac{\omega_1}{\omega_n}\\\frac{\omega_2}{\omega_1}&\frac{\omega_2}{\omega_2}&\cdots&\frac{\omega_2}{\omega_n}\\\vdots&\vdots&\ddots&\vdots\\\frac{\omega_n}{\omega_1}&\frac{\omega_n}{\omega_2}&\cdots&\frac{\omega_n}{\omega_n}\end{array}\right.$$

- Step 4: Check the reliability of the results by verifying the CR (Consistency Ratio) of the decision-making factors, and set priorities based on relative weights. Since the AHP analysis is based on the subjective opinion of experts, the CR should be verified to judge the reliability of the results. In general, if the CR is less than 10%, it is judged to be reliable. If not, re-analysis should be performed. Based on the relative weight of reliable decision-making factors, priorities are determined, and the best alternative is derived.

### Data collection

In AHP analysis, the pairwise comparison is performed with factors in the same hierarchy to confirm the relative importance of attributes that form the hierarchy of decision-making. In order to conduct a pairwise comparison, it is necessary to have direct experience in doctor-patient communication or to be an expert with sufficient knowledge and theory, as well as to have the objectivity to evaluate it. In particular, in the case of doctor-patient communication, it was judged that important factors would come out differently depending on the group, so the subjects were recruited by dividing them into medical staff and general patients. In the case of medical staff, 15 doctors working in primary medical institutions and tertiary general hospitals were selected. In the case of patients, 20 patients who majored in healthcare and had experience in hospital treatment were selected for objective judgment. All of the subjects had sufficient knowledge about health communication and medical service provision, so it was judged that they were suitable for study subjects. In other words, in this study, a survey was conducted targeting experts and stakeholders related to doctor-patient communication and a pairwise comparison was performed. Based on these criteria, a total of 35 questionnaires were distributed; 21 responses were obtained through the online survey. At this time, consent was obtained from all respondents prior to the questionnaire, and a survey was conducted derive factors that reinforcement doctor-patient communication. In addition, the main category and selection criteria were explained to the respondents, and they were asked to respond in consideration of their influence. The survey was conducted from January 13 to February 3, 2020. Additional information on the questionnaire items is presented in Supplementary File 1.

AHP is an integrated methodology that enables decision-makers to make correct decisions by using empirical data such as previous research and the subjective opinions of experts. It is also a systematic approach developed to derive solutions in a priority order to solve a particular problem [29]. In other words, in AHP, it is important to determine which factor is the priority among numerous factors for problem-solving. This has the disadvantage that it may vary depending on the individual preferences of the research subjects, so the logical consistency of the results is judged by calculating the CR for the analysis results. Since the purpose of AHP analysis is to derive valid and consistent judgments from study subjects who have professional knowledge or experience on a given problem, there is no set standard for an appropriate standard. Also, according to previous studies, the weighted sum of errors was the smallest when the sample size was 6–8 people, and it was considered that consistency could be judged when there were at least 7 people [30, 31].

In addition, looking at previous research using AHP analysis, Kip, M. et al. (2019) reviewed the questionnaires of 12 related experts in order to understand the priority of factors that determine the implementation of POC (Point-Of-Care) tests, of which 10 people were subjected to an AHP survey [32]. See also Şahin, T. et al. (2019) conducted an AHP analysis using 15 faculty in health management, health administration and policy fields to select the optimal site for establishing a new hospital [33]. Mumtaz, A. (2022) conducted an AHP analysis of 10 experts including scholars and policymakers to prioritize were identified the barriers felt by elderly people in using e-Health [34]. Most of the previous research using AHP analysis selected a sample size of around 10 to 15 people, and the sample size of this study(*n* = 21) is considered appropriate for quantitative analysis, as there are more than in the previous study.

The data collected through our survey were analyzed using DRESS (1.7.00, CHOISH Software DRESS, Korea)—the solution exclusively for an AHP. The study subjects included 7 doctors and 14 patients. Examining the general characteristics of the study subjects, they mostly consisted of females, or more specifically, 4 males (19.0%), and 17 females (81.0%). In addition, most were in their 20 and 30 s, accounting for 76.2% of the subjects. In terms of occupation, 5 were students (23.85), 9 were office workers (42.9%), and 7 were doctors (33.3%). Meanwhile, 19 subjects (90.5%) had experienced medical treatments, whereas 2 subjects (9.5%) had not, but study subjects without medical treatment experience comprised physicians, who were judged to be suitable for an analysis (See Table 4).

**Table 4:** Demographic information

Characteristic	Frequency among subjects (n)	%
Gender		
Male	4	19.0
Female	17	81.0
Age		
20s	8	38.1
30s	8	38.1
40s	4	19.0
50s	1	4.8
Job		
Student	5	23.8
Worker	9	42.9
Doctor	7	33.3
Medical experience		
Yes	19	90.5
No	2	9.5

## Results

### Comparative analysis of key factors in health communication

Priorities were derived by dividing the main factors of health communication into medical staff and patients. The CR of the doctor and patient groups, the subjects of this study, was 0.01, suggesting the reliability of the study results. In the case of doctors, the priority of health communication was in the order of professionalism (0.39), reliability (0.38), fairness (0.10), mutual intelligibility (0.09), and psychologically (0.04). On the other hand, the priority of the patient’s health communication appeared in the order of mutual intelligibility (0.34), fairness (0.20), professionalism (0.19), reliability (0.17), and psychologically (0.10), showing that doctors and patients have different priorities for health communication. That is, in the case of doctors, professionalism and reliability were found to be the main factors of health communication, while in terms of patients, mutual intelligibility and fairness appeared to be the main factors of health communication (See Table 5).

**Table 5:** Analysis of key factors

Top Item	Factor Weights					
	Medical staff			Patient		
	Importance	Priority	Consistency Ratio	Importance	Priority	Consistency Ratio.
Reliability	0.38	2	0.01	0.17	4	0.01
Communication	0.09	4		**0.34**	**1**	
Professionalism	**0.39**	**1**		0.19	3	
Psychologically	0.04	5		0.10	5	
Fairness	0.10	3		0.20	2	
Total	1.0			1.0		

### Comparative analysis of health communication

Priorities were derived by classifying the sub-items of health communication to improve the quality of medical care for medical staff and patients. The priority of sub-items for the doctor’s reliability appeared in the order of conformity of words and actions (0.49), sincere participation in treatment (0.28), and provision of accurate information (0.23). In the case of mutual intelligibility, it appeared in the order of treatment through consultation (0.38), checking whether the treatment content is perceived (0.34), and collecting opinions (0.28). Furthermore, in the case of professionalism, it was in the order of accuracy of diagnosis and prescription (0.48), professional medical knowledge and the latest information (0.39), and the patient’s high cognition (0.13). In psychologically, it appeared in the order of creating a comfortable atmosphere (0.42), trusting and relying on doctors (0.39), and doctor’s kindness (0.18). Lastly, in the case of fairness, it was in the order of respecting patients (0.47), consistency of treatment action (0.42), and ease of providing treatment information (0.11).

On the other hand, when examining the patient’s health communication priority, in the case of reliability, it was found in the order of conformity of words and actions (0.39), provision of accurate information (0.38), and sincere participation in treatment (0.23). In addition, for mutual intelligibility, it appeared in the order of checking whether the treatment content was perceived (0.50), treatment through consultation (0.28), and collecting opinions (0.22). In the case of professionalism, it was in the order of accuracy of diagnosis and prescription (0.58), professional medical knowledge and the latest information (0.27), and patient’s high cognition (0.15). For psychologically, it was found in the order of creating a comfortable atmosphere (0.44), trusting and relying on doctors (0.29), and doctor’s kindness (0.27). Lastly, in the case of fairness, it appeared in the order of ease of providing treatment information (0.38), consistency of treatment action (0.31), and respecting patients (0.31). In other words, in terms of professionalism and psychologically, the priority of the sub-items for health communication between doctors and patients was found to be consistent for both medical staff and patients, while the sub-items of reliability, mutual intelligibility, and fairness appeared to differ from each other (See Table 6).

**Table 6:** Medical staff–patient health communication priority analysis

Top Item	Sub-item	Factor Weights					
		Medical staff			Patient		
		Importance	Priority	CR	Importance	Priority	CR
Reliability	Consistent in words and actions	**0.49**	**1**	0.03	**0.39**	**1**	0.01
	Provides accurate information	0.23	3		0.38	2	
	Patient’s sincere participation	0.28	2		0.23	3	
Communication	Respect patients’ opinions	0.28	3	0.06	0.22	3	0.06
	Discusses the treatment process with the patient	**0.38**	**1**		0.28	2	
	Confirms that the patient has recognized	0.34	2		**0.50**	**1**	
Professionalism	Diagnosis and prescription accuracy	**0.48**	**1**	0.00	**0.58**	**1**	0.00
	Possesses professional medical knowledge and the latest information	0.39	2		0.27	2	
	Patient’s high cognition	0.13	3		0.15	3	
Psychologically	Creates a comfortable atmosphere	**0.42**	**1**	0.00	**0.44**	**1**	0.00
	Trusts and relies on the doctor	0.39	2		0.29	2	
	Doctor’s kindness	0.18	3		0.27	3	
Fairness	Consistency in the treatment process	0.42	2	0.00	0.31	2	0.01
	Patient respect	**0.47**	**1**		0.31	2	
	Easily receives treatment information	0.11	3		**0.38**	**1**	
Total		4.0			4.0		

## Discussion

In this study, we intended to suggest measures for establishing positive relationships between doctors and patients by uncovering differences revealed in doctor–patient communication and then resolving these. As a result of this study, for physicians, we consider professionalism as an important factor in health communication. Professionalism is a core competency of doctors, who are responsible for providing medical services; doctors’ professionalism acts as an important part of building trust between doctors and patients, which was found to be consistent with existing research findings [23]. Since doctors are expected to provide accurate diagnoses for patients, doctors largely focus on clearly communicating the results of diagnoses to the patients, rather than emotionally empathizing with them.

In addition, among the sub-items of professionalism in this study, the accuracy of diagnoses and prescriptions appeared as the top item, similar to the results of previous studies that have stated that state-of-the-art medical knowledge, high-level clinical competence, and communication skills are key elements necessary to represent the doctor’s professionalism [26, 27]. For health communication, studies have simply defined doctors’ professionalism as having clinical skills and expert knowledge and believe that what the physicians do is playing a role in instructing patients about diagnosis and treatment recommendations, regardless of patient preference [28]. However, in recent years, the definition of doctors’ professionalism has included, among others, a kind attitude toward the patient and polite speech, which also acts as an important factor, even in terms of the quality of medical services. Patient satisfaction is induced by the professionalism of the healthcare provider, and this professionalism includes a specific explanation of the treatment period and the smooth communication with the medical staff, among others [35].

In the case of patients, unlike doctors, we considered that the most important factor in health communication was mutual intelligibility. This was found to be similar to the conclusions of previous studies that patients generally desire to share their thoughts on the causes of their diseases with doctors. Thus, the communication skills of doctors may form a positive doctor–patient relationship that would eventually lead to an improvement in treatment results [36, 37]. The communication styles of doctors can be divided into doctor-centered and patient-centered styles. The doctor-centered style is a communication style in which a doctor unilaterally directs information on treatment and can be seen as communication that focuses only on treatment. On the other hand, the patient-centered style indicates that doctors and patients communicate with each other; in this case, the patient’s accepting attitude is shown to be active. Accordingly, it is judged that the quality of medical care services could be improved if the information necessary for treatment is obtained from patients through patient-centered health communication, and then mutually cooperative relationships could be established.

In addition, the patients selected checking whether the treatment content is perceived as the top factor among the sub-items of mutual-intelligibility. These results were found to differ from existing study results that negative interactions between doctors and patients are mainly induced by nonverbal communication errors and that non-verbal communication, including the coldness of the doctor’s facial expressions and the doctor’s emotional message indicating boredom for the patient’s explanations, is the main cause of lower patient satisfaction with medical services [13]. After the doctor informs the treatment process to the patient through diagnosis, it seems necessary for the physician to receive feedback from the patient with respect to the treatment process—for example, whether it was understood by the patient or whether the patient had any questions, among others. Furthermore, when a doctor and patient build a sense of trust between each other, the patient will naturally express his or her feelings of needs, expectations, and inconveniences to the doctor. This sense of trust can be formed when doctors utilize nonverbal communication, such as through expressing their sympathy for the patient’s words, and so forth. Through a doctor’s body language, such as their facial expressions and postures, which the patient comes to confront after entering the examining room, the patient would form a rapport with the doctor and explain his or her conditions in detail [23, 38]. Therefore, a physician needs to judge a patient’s individual situation, to present a treatment process appropriate for the patient, and to check whether the patient understands it through sufficient explanations so that the patient may carry it out. For doctors to effectively communicate a treatment process to patients, mutual understanding centered on communication must be the basis, and smooth health communication would be able to improve the quality of medical care and safety in the treatment process.

In the meantime, doctors selected reliability as the top factor in health communication, whereas patients selected fairness. It was found that this differs from existing study findings that patients are able to trust doctors when physicians show respectful manners and sympathizing attitudes to them, and thereby, the quality of communication could also be improved [39, 40]. Fairness implies that a doctor will fairly treat all patients, regardless of their social status or personal relationships. It seems that doctors’ discrimination against patients according to the latter’s social status, personal closeness, and personal connections can interfere with the relationship between patients and doctors. Physicians need to work hard to make patients better understand their own disease status, without the latter feeling alienated. Through this, it is judged that if treatment is performed from the patient’s point of view by resolving the potential misunderstanding that the relationship would be unfair between doctors and patients, it would be of great help in improving the outcome of treatment from a long-term perspective. On the other hand, doctors viewed reliability as the top factor in health communication. Doctors believe that clinical skills are an important part of communication behavior with patients and consider it an important factor in giving patients a sense of trust based on counseling to understand the patients’ medical history, explaining diagnoses and prognoses, and providing accurate treatment methods, among others. Patients complain that they tend to lose their trust for doctors because they cannot receive satisfactory medical services due to the physician’s actions of unilaterally providing treatment information and instructing treatment methods, among others, without being aware of the patient’s situation. Therefore, to increase reliability for patients, doctors should be able to improve the quality of medical service by sharing the information they have regarding monitoring during the diagnosis and treatment processes, through sufficient conversations with the patient, to obtain accurate information necessary for the patient.

In this study, we presented factors that interfere with health communication and suggested measures to resolve these, from the viewpoints of both doctors and patients. However, recently, telemedicine treatment has been performed for chronic disease management using mobile medical devices, and untact healthcare due to COVID-19 are increasing. It is expected that the important factors of health communication would differ according to face-to-face and untact healthcare. In future studies, if the factors that hinder the communication between doctors and patients are identified through an additional analysis on this point, it is expected that the service quality of continuously increasing untact healthcare would be improved.

## Conclusion

In this study, doctors judged that professionalism and reliability were more important than fairness and mutual intelligibility in communication with patients, and patients considered mutual intelligibility and fairness to be more important than professionalism and reliability. To establish a positive relationship between doctor and patient, while communicating with the patient centered on his or her treatment, a doctor needs to have regular conversations about whether the patient understands what he or she is about to go through and whether the patient is in an environment where the treatment process can be performed. Patients should accurately describe their disease status to increase the accuracy of diagnoses and participate in the treatment process by continuously exchanging opinions with their doctors. To enhance health communication between doctors and patients, doctors should reinforce communication with patients by communicating from the patient’s point of view and by providing consistent medical services; patients should be able to reinforce communication with doctors by trusting doctors and faithfully participating in the treatment process. Based on this, if patient-centered medical services are provided, it is judged that a successful treatment strategy can be established based on the patient’s personal preference.

## Supplementary Information

**Additional file 1: Appendix 1.** Questionnaire used in the research.

## Data Availability

The datasets used and analysed during the current study are available from the corresponding author on reasonable request.

## Abbreviations

AHP: Analytic Hierarchy Process
CR: Consistency Ratio
MCDM: Multi-Criteria Decision Making
POC: Point-Of-Care
