# Supplementary material for: An analytic hierarchy process analysis for reinforcing doctor–patient communication
Source: BMC Prim Care. 2023 Jan 21;24:24. doi: 10.1186/s12875-023-01972-3 (PMC9860231; doi:10.1186/s12875-023-01972-3)
Supplement: Supplementary file 1 — Additional file 1: Appendix 1. Questionnaire used in the research. [file 12875_2023_1972_MOESM1_ESM.docx]

# **Supplementary Information**

Appendix 1. Questionnaire used in the research

| **① The following are five areas of the top item of health communication. Please tick (√) the degree that you think is important.** | | | | | | | | | | | | | | | | | | |
| --- | --- | --- | --- | --- | --- | --- | --- | --- | --- | --- | --- | --- | --- | --- | --- | --- | --- | --- |
| **Top Item (A)** | **A Is Important** | | | | | | | | **1** | **B Is Important** | | | | | | | | **Top Item (B)** |
|  | **9** | **8** | **7** | **6** | **5** | **4** | **3** | **2** |  | **2** | **3** | **4** | **5** | **6** | **7** | **8** | **9** |  |
| Reliability |  |  |  |  |  |  |  |  |  |  |  |  |  |  |  |  |  | Communication |
| Reliability |  |  |  |  |  |  |  |  |  |  |  |  |  |  |  |  |  | Professionalism |
| Reliability |  |  |  |  |  |  |  |  |  |  |  |  |  |  |  |  |  | Psychologically |
| Reliability |  |  |  |  |  |  |  |  |  |  |  |  |  |  |  |  |  | Fairness |
| Communication |  |  |  |  |  |  |  |  |  |  |  |  |  |  |  |  |  | Professionalism |
| Communication |  |  |  |  |  |  |  |  |  |  |  |  |  |  |  |  |  | Psychologically |
| Communication |  |  |  |  |  |  |  |  |  |  |  |  |  |  |  |  |  | Fairness |
| Professionalism |  |  |  |  |  |  |  |  |  |  |  |  |  |  |  |  |  | Psychologically |
| Professionalism |  |  |  |  |  |  |  |  |  |  |  |  |  |  |  |  |  | Fairness |
| Psychologically |  |  |  |  |  |  |  |  |  |  |  |  |  |  |  |  |  | Fairness |
| **② This is a sub-item for reliability. Please tick (√) the degree you think is important as a sub-item of reliability.** | | | | | | | | | | | | | | | | | | |
| **Top Item (A)** | **A Is Important** | | | | | | | | **1** | **B Is Important** | | | | | | | | **Top Item (B)** |
|  | **9** | **8** | **7** | **6** | **5** | **4** | **3** | **2** |  | **2** | **3** | **4** | **5** | **6** | **7** | **8** | **9** |  |
| Consistent in words and actions |  |  |  |  |  |  |  |  |  |  |  |  |  |  |  |  |  | Provides accurate information |
| Consistent in words and actions |  |  |  |  |  |  |  |  |  |  |  |  |  |  |  |  |  | Patient’s sincere participation |
| Provides accurate information |  |  |  |  |  |  |  |  |  |  |  |  |  |  |  |  |  | Patient’s sincere participation |
| **③ This is a sub-item for communication. Please tick (√) the degree you think is important as a sub-item of communication.** | | | | | | | | | | | | | | | | | | |
| **Top Item (A)** | **A Is Important** | | | | | | | | **1** | **B Is Important** | | | | | | | | **Top Item (B)** |
|  | **9** | **8** | **7** | **6** | **5** | **4** | **3** | **2** |  | **2** | **3** | **4** | **5** | **6** | **7** | **8** | **9** |  |
| Respect patients’ opinions |  |  |  |  |  |  |  |  |  |  |  |  |  |  |  |  |  | Discusses the treatment process with the patient |
| Respect patients’ opinions |  |  |  |  |  |  |  |  |  |  |  |  |  |  |  |  |  | Confirms that the patient has recognized |
| Discusses the treatment process with the patient |  |  |  |  |  |  |  |  |  |  |  |  |  |  |  |  |  | Confirms that the patient has recognized |
| **④ This is a sub-item for professionalism. Please tick (√) the degree you think is important as a sub-item of professionalism.** | | | | | | | | | | | | | | | | | | |
| **Top Item (A)** | **A Is Important** | | | | | | | | **1** | **B Is Important** | | | | | | | | **Top Item (B)** |
|  | **9** | **8** | **7** | **6** | **5** | **4** | **3** | **2** |  | **2** | **3** | **4** | **5** | **6** | **7** | **8** | **9** |  |
| Diagnosis and prescription accuracy |  |  |  |  |  |  |  |  |  |  |  |  |  |  |  |  |  | Possesses professional medical knowledge and the latest information |
| Diagnosis and prescription accuracy |  |  |  |  |  |  |  |  |  |  |  |  |  |  |  |  |  | Patient’s high cognition |
| Possesses professional medical knowledge and the latest information |  |  |  |  |  |  |  |  |  |  |  |  |  |  |  |  |  | Patient’s high cognition |
| **⑤ This is a sub-item for psychologically. Please tick (√) the degree you think is important as a sub-item of psychologically.** | | | | | | | | | | | | | | | | | | |
| **Top Item (A)** | **A Is Important** | | | | | | | | **1** | **B Is Important** | | | | | | | | **Top Item (B)** |
|  | **9** | **8** | **7** | **6** | **5** | **4** | **3** | **2** |  | **2** | **3** | **4** | **5** | **6** | **7** | **8** | **9** |  |
| Creates a comfortable atmosphere |  |  |  |  |  |  |  |  |  |  |  |  |  |  |  |  |  | Trusts and relies on the doctor |
| Creates a comfortable atmosphere |  |  |  |  |  |  |  |  |  |  |  |  |  |  |  |  |  | Doctor’s kindness |
| Patient’s high cognition |  |  |  |  |  |  |  |  |  |  |  |  |  |  |  |  |  | Doctor’s kindness |
| **⑥ This is a sub-item for fairness. Please tick (√) the degree you think is important as a sub-item of fairness.** | | | | | | | | | | | | | | | | | | |
| **Top Item (A)** | **A Is Important** | | | | | | | | **1** | **B Is Important** | | | | | | | | **Top Item (B)** |
|  | **9** | **8** | **7** | **6** | **5** | **4** | **3** | **2** |  | **2** | **3** | **4** | **5** | **6** | **7** | **8** | **9** |  |
| Consistency in the treatment process |  |  |  |  |  |  |  |  |  |  |  |  |  |  |  |  |  | Patient respect |
| Consistency in the treatment process |  |  |  |  |  |  |  |  |  |  |  |  |  |  |  |  |  | Easily receives treatment information |
| Patient respect |  |  |  |  |  |  |  |  |  |  |  |  |  |  |  |  |  | Easily receives treatment information |
